# Nucleo-cytoplasmic interactions affecting biological performance of *Lipaphis erysimi* in *Brassica juncea*

**DOI:** 10.3389/fpls.2022.971606

**Published:** 2022-08-18

**Authors:** Naveen Singh, Mukesh K. Dhillon

**Affiliations:** ^1^Division of Genetics, ICAR-Indian Agricultural Research Institute, New Delhi, India; ^2^Division of Entomology, ICAR-Indian Agricultural Research Institute, New Delhi, India

**Keywords:** *Brassica juncea*, *Lipaphis erysimi*, cytoplasmic male sterility, host plant resistance, mustard aphid, resistance mechanisms

## Abstract

Hybrids have been successfully used to improve crop productivity, including Brassicas. Nucleo-cytoplasmic interactions have been reported to influence the expression of resistance to insect pests in several crops. We studied the effects of Cytoplasmic Male Sterility (CMS) in *Brassica juncea* carrying alien cytoplasms and their respective maintainer (B) lines on the antibiosis mechanism of resistance, involving development, survival, reproduction potential and population build-up of mustard aphid, *Lipaphis erysimi*, and the levels of defense phyto-chemicals. Present findings revealed that the numbers of aphids/plant, aphid multiplication rate and aphid resistance index were lower on *ber* CMS under natural, *mori* CMS under artificial infestation conditions, and *juncea* under both the test conditions indicating nucleo-cytoplasmic interactions for aphid reaction. Across cytoplasms, nymphal, reproductive and total developmental periods were significantly longer on SEJ 8, NPJ 161, LES 39, and NPJ 93, while the reproductive potential and survival were lower on PM 30, Pusa Tarak and SEJ 8 as compared to other nuclear backgrounds. Across nuclear backgrounds, nymphal, reproductive and total developmental periods were significantly longer on *ber* CMS, while reproductive potential and survival were lower on *ber* and *mori* CMS as compared to other cytoplasms. Total glucosinolates were significantly greater and myrosinase lower in Pusa Agrani, SEJ 8, LES 39, PM 30, NPJ 112, and Pusa Tarak as compared to the other nuclear backgrounds. Furthermore, total glucosinolates were significantly greater and myrosinase lower in *ber* CMS and *juncea* as compared to other cytoplasms. The studies suggest that CMS as well as cytoplasmic and nuclear gene interactions regulate the expression of defense compounds such as glucosinolates and determine the expression of resistance/susceptibility to *L. erysimi*. These findings shall help in identification of suitable *L. erysimi* tolerant nucleo-cytoplasmic combinations for their deployment in *B. juncea* hybrid breeding program.

## Introduction

Rapeseed-mustard (*Brassica* spp.) is the third most important oilseed crop after soybean and oil palm (Palial et al., 2022), and contributes 27.8% to the Indian oilseed industry (Kumrawat and Yadav, 2018). Among the oilseed brassicas, *Brassica juncea* (L.) Czern & Coss. occupies > 90% acreage in the country (Singh, 2014). The average productivity of mustard in India (1,511 kg/ha) is much lower than the world average (1,980 kg/ha), which is about three-fourths of the world average (Soybean Processors Association of India [SOPA], 2020). Further, the production and productivity of rapeseed-mustard are highly variable due to various biotic and abiotic stresses experienced across crop-growing agro-ecologies of India. Among the biotic stresses, mustard aphid, *Lipaphis erysimi* (Kaltenbach) is the major yield reducing factor in rapeseed-mustard, causing up to 90% yield loss under severe infestation conditions (Ahuja et al., 2010).

To meet the rising global food demand, hybrids have been successfully used to improve the productivity across the crops, including Brassicas. Cytoplasmic-genetic male sterility has been one of the most efficient hybrid seed production systems deployed for commercial exploitation of heterosis phenomenon in a wide array of field crops (Dhillon et al., 2008). The cytoplasmic male sterility and fertility restorer (CMS-FR) system, comprising of male sterile (A), maintainer (B), and fertility restorer (R) lines, has been extensively used both in cross- and self-pollinated crop species for commercial hybrid seed production (Melonek et al., 2021).

Several male-sterility inducing cytoplasmic sources have been identified among wild or related species of *Brassica* and nuclear genome of cultivated Brassicas has been transferred through intergeneric or interspecific hybridization into these sterility-inducing cytoplasms such as *Raphanus sativus* (*ogu*; Kirti et al., 1995), *B. tournefortii* (*tour*; Banga et al., 1995; Pahwa et al., 2004), *Moricandia arvensis* (*mori*; Kirti et al., 1998; Prakash et al., 1998), *Diplotaxis catholica* (*cath*; Pathania et al., 2003), *Enarthrocarpus lyratus* (*lyr*; Banga et al., 2003; Janeja et al., 2003), *Diplotaxis erucoides* (*eru*; Malik et al., 1999; Bhat et al., 2006), and *Diplotaxis berthautii* (*ber*; Malik et al., 1999; Bhat et al., 2008). Among these, *ogu* and *mori* CMS systems are widely used for development of commercial *B. juncea* hybrids in India (Kaur et al., 2004). Furthermore, the common fertility restorer (*Rf*) gene from *mori* restores fertility in *mori*, *eru*, and *ber* cytoplasms (Bhat et al., 2005, 2006, 2008), and offers an opportunity to diversify cytosterility sources without the need of searching for an appropriate restorer gene (Vinu et al., 2017). Good number of test hybrids with *eru*, *ber*, and *mori* cytoplasms have been developed at the Indian Council of Agricultural Research-Indian Agricultural Research Institute (ICAR-IARI), New Delhi (Chamola et al., 2013), and have been evaluated in multilocation trials by the All India Coordinated Research Project on Rapeseed-Mustard (Anonymous, 2021).

Large-scale cultivation of crop hybrids carrying one or a few CMS sources may lead to decrease in genetic diversity, and make them vulnerable to various pests over time and adversely impact sustainable crop production (Dalmacio et al., 1995). Therefore, diversification of cytoplasmic as well as nuclear genome of parental lines is important for sustainable hybrid breeding programs in rapeseed-mustard (Vinu et al., 2017). Furthermore, information available on different CMS systems in cereals revealed that the nuclear background of CMS, cytoplasmic factors, and the interaction effects of cytoplasm and nuclear genomes influence the expression of resistance to insect pests and pathogens (Sharma et al., 2004; Dhillon et al., 2006b; Dhillon et al., 2008). However, information on the effects of sterility inducing cytoplasms on the expression of resistance/susceptibility reaction in rapeseed-mustard against *L. erysimi* is still missing. Thus, present studies were carried out to decipher the effects of different CMS systems (A lines) and the respective maintainers (B lines) of *B. juncea* on the development, survival, reproduction potential and population build-up of *L. erysimi*, and on the levels of major plant defense biochemicals like glucosinolates and myrosinase. For this purpose, a set of isonuclear alloplasmic lines in different *B. juncea* backgrounds was developed at ICAR-IARI following backcross breeding. The relative performance of these isonuclear alloplasmic lines, carrying cytoplasms from wild species, against *L. erysimi* shall help in identification of suitable cytoplasmic sources and their deployment in *B. juncea* hybrid breeding program.

## Materials and methods

### Plant material

The experimental material consisted of 11 diverse *B. juncea* maintainer (B) lines and 33 CMS (A) lines carrying three different sterility-inducing cytoplasms *viz.*, *D. berthautii* (*ber*), *D. erucoides* (*eru*) and *M. arvensis* (*mori*) in each maintainer background. Thus, making a set of 33 CMS lines and their 11 *B. juncea* nuclear donors. The CMS lines and their respective maintainers were sown in 4 row plots of 5 m row length keeping 45 cm row to row and 15 cm plant to plant distance during *rabi* (winter) 2020–2021 season in the experimental fields of ICAR-IARI, New Delhi. All recommended agronomic practices, except insecticide use, were followed to raise the crop.

### Evaluation of A and B lines of *Brassica juncea* for aphid multiplication rate and resistance index

#### Natural infestation

To observe the natural infestation of aphids in the A and B lines, five plants were randomly selected from each plot and labeled, thus making five replications in a randomized block design. The observations were recorded on the number of aphids, aphid population index (API on a rating scale of 1–5) and aphid damage index (ADI on a rating scale of 1–5) of all the test genotypes as described by Dhillon et al. (2018). The A and B lines were monitored daily to track mustard aphid infestation, and economic threshold level (ETL; 15 aphids on top 10 cm twig in 10% of plants). After 15 days when *L. erysimi* reached ETL, the number of aphids on the main branch (upper 10 cm) and aphid resistance index was computed (API + ADI/2) from the labeled plants of each A and B lines as described by Dhillon et al. (2018).

#### Artificial infestation

Five randomly selected plants of each A and B lines were tagged for artificial infection, thus making five replications in a completely randomized design. At the bud initiation stage, around 20 aphids (nymphs and adults) were inoculated with pieces of infested *Brassica* twigs pinned on the third shoot from the top of the plant. After aphid inoculation in the buds, the branch was covered with the specially designed twig cage as described by Dhillon et al. (2018). At 15 days after *L. erysimi* inoculation, observations were recorded on total number of aphids on the caged shoot of each tagged plant. The aphid multiplication rate was obtained by dividing the total number of aphids by 20 for each test plant, and A and B lines separately.

### Developmental period, reproductive potential and survival of *Lipaphis erysimi* on A and B lines of *Brassica juncea*

The biological performance studies of *L. erysimi* on different plant parts, *viz*, leaves, buds, and siliquae of the A and B lines carrying *B. juncea* nuclear genome, were carried out under laboratory conditions at 17 ± 3*^o^* C temperature, 60–70% relative humidity and 12L:12D photoperiod. The *L. erysimi* were collected from the field and reared on mustard leaves in glass Petri dishes (10 cm h × 2 cm dia.) under laboratory conditions. The moistened filter paper was placed inside the Petri dish. The test plant parts *viz.*, leaf discs, buds and siliquae of each test line were placed on the moist blotting paper to keep them turgid. The newly produced nymphs obtained from the laboratory-reared aphids were transferred to the test plant parts (one nymph each) with the help of a fine moist camel hairbrush. There were 15 replications, for each line and plant part, in a completely randomized design. The respective plant parts were changed daily till the completion of the studies. The observations were recorded on total nymphal period, reproductive period, total developmental period, reproductive potential and survival of *L. erysimi*. The observations were recorded at 12 h intervals. The total nymphal period was calculated as the period (in hours) between the start of the first instar to the end of the fourth instar stage. The reproductive period of an individual aphid was recorded as the duration (in hours) between it giving birth to the first and the last nymph. The total developmental period was calculated as the time period (in hours) between the birth of the first instar till its death as an adult. The reproductive potential, i.e., number of nymphs produced by each female during its reproductive period was recorded, and expressed as nymphs/female. The observations were recorded till the death of an adult. The total nymphs produced by each female were counted and per cent survival of nymphs was calculated till 48 h, and expressed as per cent survival/female.

### Estimation of glucosinolates and myrosinase in different plant parts of A and B lines of *Brassica juncea*

#### Glucosinolates

For glucosinolate quantification, fresh leaves, buds and siliquae samples from the test genotypes were collected and oven dried at 70°C, then 300 ml of 80% methanol was added to 0.3 g of dried sample, and incubated at 70°C for 5 min. After cooling, 2 mL double distilled water was added to the mixture, and the samples were centrifuged at 15,000 rpm for 15 min, and after that, the supernatants were collected for further analysis. After adding 100 μl of supernatant and 4 ml of 0.2 Mm sodium tetrachloropalladate, the mixture was incubated at room temperature for 1 h. Test samples (200 μl) were dispensed into each well of a 96-well microtiter plate, and absorbance was recorded at a wavelength of 425 nm in an ELISA Reader (SPECTRA max Plus 384, United States). Total glucosinolate content was calculated by using the OD value of each sample in the formula [*y* = 1.40 + 118.86 × A_425_] given by Mawlong et al. (2017), and the values thus obtained were expressed as μ moles/g of plant tissue.

#### Myrosinase

For myrosinase estimation, 50 mg tissue samples of leaves, buds or siliquae were homogenized in 1 ml distilled water and centrifuged at 13,000 rpm for 5 min at 4°C, and the supernatant was collected. The supernatant (15 μl) was mixed with 1,470 μl of 80 mM NaCl (pH 6.5) and 15 μl of 20 mM sinigrin and incubated at 37°C (Piekarska et al., 2013). The myrosinase activity was determined based on measurements of decomposition of sinigrin by following the decrease in absorbance of the reaction mixture at 230 nm using a spectrophotometer (Thermo Fisher Scientific, United States). The total final volume of the reaction mixture was 1.5 mL and the myrosinase activity was calculated using the formula:


(1) Activity=1,000V/AVsample⋅2(A-0At)/E⋅t

Where, “V_*A*_” denotes the volume of the reaction mixture, “Vsample” denotes the volume of investigated sample, “A_0_” denotes the initial absorbance, “At” denotes the absorbance after reaction time, “t” denotes the reaction time corresponding to the initial reaction rate characterized by a linear change in absorption (min), and ‘E’ denotes the molar extinction coefficient, i.e., 7,500 for SIN, 8,870 for GTL (1/mol cm). Final myrosinase activity was expressed as mol of hydrolyzed GLS per min recalculated per 1 g of enzyme prep (U/g prep).

### Statistical analysis

The data on population build-up, multiplication rate and resistance index under artificial and natural infestation conditions, *L. erysimi* biological parameters and the biochemical parameters in different nuclear backgrounds and cytoplasms were subjected to factorial analysis using statistical software SAS^®^ version 9.2. The data on *L. erysimi* biological and plant biochemical parameters on individual plant parts as well as averaged across plant parts were also subjected to factorial analysis. The significance of differences were tested by *F*-test, and the treatment means and their interactions were compared using LSD values at *P* = 0.05.

## Results

### Population build-up, aphid multiplication rate and aphid resistance index on A and B lines of *Brassica juncea*

#### Natural infestation

**FIGURE 1:**
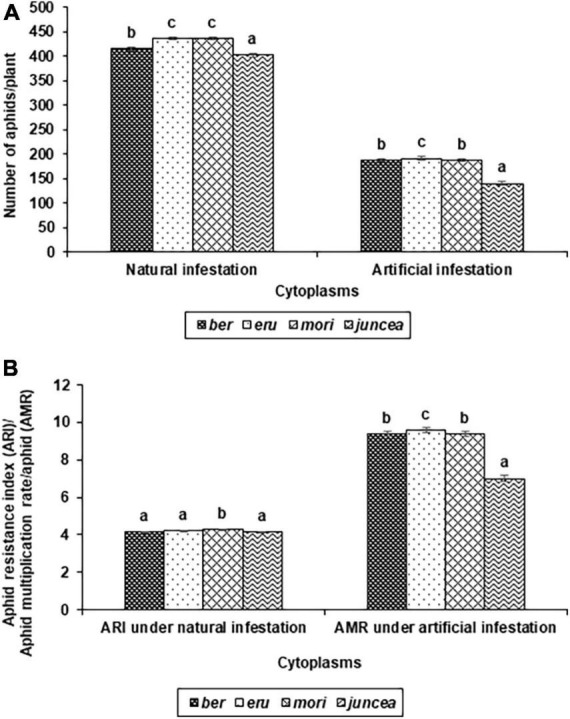
Effects of cytoplasms on population build up **(A)**, and Resistance index and multiplication rate **(B)** of *Lipaphis erysimi* under natural and artificial infestation conditions in *Brassica juncea.*

The numbers of aphids/plant and aphid resistance index on the test cytoplasms and nuclear backgrounds of *B. juncea* genotypes varied from 159 to 713 and 3.0 to 5.0 under natural infestation conditions (Table 1). There were significant differences in the number of aphids/plant and aphid resistance index among the test *B. juncea* nuclear background, cytoplasms, and the nuclear background × cytoplasm interactions (Table 1). Across cytoplasms, the numbers of aphids/plant and aphid resistance index were significantly lower on LES 39 and NPJ 93 as compared to other genotypes. Across the nuclear background, the numbers of aphids/plant (Figure 1A) and aphid resistance index (Figure 1B) were significantly lower on *ber* and *juncea* as compared to other cytoplasms. Across nuclear background and cytoplasms, the numbers of aphids/plant and aphid resistance index were significantly lower on NPJ 93 in *ber* and *mori*, LES 39 in *eru*, and SEJ 8 in *juncea* cytoplasmic backgrounds (Table 1).

**TABLE 1:** Effects of cytoplasms and nuclear backgrounds on aphid resistance index, population build up and multiplication rate of *Lipaphis erysimi* in auto- and alloplasmic lines of *Brassica juncea*.

Nuclear backgrounds (*B. juncea*)	Natural infestation conditions										Artificial infestation conditions									
	Aphids/plant (top 10 cm branch)					Aphid resistance index					Aphids/plant					Aphid multiplication rate/aphid				
	*ber*	*eru*	*mori*	*juncea*	Mean	*ber*	*eru*	*mori*	*juncea*	Mean	*ber*	*eru*	*mori*	*juncea*	Mean	*ber*	*eru*	*mori*	*juncea*	Mean
Laxmi	466.0	477.0	536.0	566.0	511.3f	4.0	4.2	5.0	5.0	4.6e	201.0	128.0	194.0	161.0	171.0e	10.1	6.4	9.7	8.1	8.6c
LES 39	364.0	213.0	401.0	360.0	334.5b	4.0	3.0	4.0	4.0	3.8a	203.8	264.0	163.0	122.0	188.2f	10.2	13.2	8.2	6.1	9.4c
NPJ 112	332.0	314.0	369.0	412.0	356.8c	4.0	4.0	4.0	4.0	4.0c	180.2	163.2	201.8	174.0	179.8e	9.0	8.2	10.1	8.7	9.0c
NPJ 139	696.0	590.0	650.0	712.6	662.2h	5.0	5.0	5.0	5.0	5.0f	182.0	130.0	97.0	85.0	123.5b	9.1	6.5	4.9	4.3	6.2a
NPJ 161	400.0	354.0	402.0	275.0	357.8c	4.0	4.0	4.0	3.8	4.0c	210.2	121.6	129.0	86.2	136.8c	10.5	6.1	6.5	4.3	6.9b
NPJ 93	210.4	404.0	159.0	364.0	284.4a	3.0	4.0	3.0	4.0	3.5a	95.0	143.2	131.8	122.4	123.1b	4.8	7.2	6.6	6.1	6.2a
PM 30	318.0	536.0	327.0	277.0	364.5c	4.1	5.0	4.0	4.0	4.3d	127.8	98.0	100.0	101.0	106.7a	6.4	4.9	5.0	5.1	5.4a
Pusa Agrani	435.0	464.0	422.0	271.0	398.0d	4.0	4.0	4.0	3.8	4.0c	151.0	81.0	129.6	189.6	137.8c	7.6	4.1	6.5	9.5	6.9b
Pusa Kisan	537.0	527.0	529.0	592.0	546.3g	5.0	5.0	5.0	5.0	5.0f	370.0	580.0	550.0	212.0	428.0g	18.5	29.0	27.5	10.6	21.4e
Pusa Tarak	282.0	489.0	468.0	338.0	394.3d	3.6	4.0	4.0	4.0	3.9b	211.0	219.0	240.0	111.8	195.5f	10.6	11.0	12.0	5.6	9.8d
SEJ 8	522.0	432.0	534.0	250.0	434.5e	5.0	4.0	5.0	3.2	4.3d	135.0	175.4	132.0	172.8	153.8d	6.8	8.8	6.6	8.6	7.7b
Mean	414.8b	436.4c	436.1c	401.6a		4.2a	4.2a	4.3b	4.2a		187.9b	191.2b	188.0b	139.8a		9.4a	9.6a	9.4a	7.0b	
For comparing	F-probability		LSD (*P* = 0.05)			F-probability		LSD (*P* = 0.05)			F-probability		LSD (*P* = 0.05)			F-probability		LSD (*P* = 0.05)		
Genotypes (G)	<0.001		14.68			<0.001		0.10			<0.001		14.97			<0.001		0.75		
Cytoplasm (C)	<0.001		8.85			<0.001		0.06			<0.001		9.03			<0.001		0.45		
G × C	<0.001		29.36			<0.001		0.20			<0.001		29.94			<0.001		1.5		

The mean values in a row for a parameter following different letters are significant at P = 0.05. The mean values in a column for a parameter following different letters are significant at P = 0.05.

#### Artificial infestation

The numbers of aphids/plant and aphid multiplication rate on the tested cytoplasms across nuclear backgrounds of *B. juncea* varied from 81 to 580 and 4.1 to 29.0 under artificial infestation conditions (Table 1). There were significant differences for the number of aphids/plant and aphid multiplication rate among the test *B. juncea* nuclear backgrounds, cytoplasms, and the nuclear background × cytoplasm interactions (Table 1). Across cytoplasms, the numbers of aphids/plant and aphid multiplication rate were significantly lower on PM 30, Pusa Agrani, NPJ 93, NPJ 139, NPJ 161, and SEJ 8 as compared to other genotypes. Across nuclear backgrounds, the numbers of aphids/plant (Figure 1A) and aphid multiplication rate (Figure 1B) were significantly lower on *juncea* as compared to other (*ber, eru* and *mori*) cytoplasms. Among male sterile cytoplasms, it was comparatively lower on *ber* and *mori* as compared to *eru* cytoplasm (Figures 1A,B). Across nuclear backgrounds and cytoplasms, the numbers of aphids/plant and aphid multiplication rate were significantly lower on NPJ 93 in *ber*, Pusa Agrani in *eru*, NPJ 139 in *mori*, and NPJ 161 and NPJ 139 in *juncea* cytoplasmic backgrounds (Table 1).

### Developmental period, reproductive potential and survival of *Lipaphis erysimi* on A and B lines of *Brassica juncea*

#### Nymphal period

**FIGURE 2:**
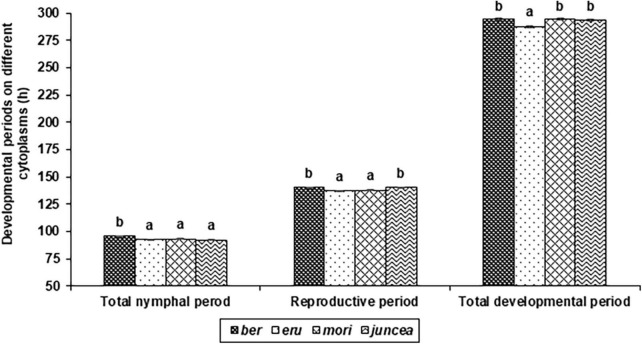
Effects of cytoplasms on developmental and reproductive periods of *Lipaphis erysimi* in *Brassica juncea.*

The total nymphal period of *L. erysimi* varied from 75.2 to 104.2 h on the leaves, 83.5 to 112.0 h on buds and 83.0 to 114.6 h on the siliquae, and there were significant differences among the test *B. juncea* nuclear backgrounds, across cytoplasms, and the nuclear background × cytoplasm interactions (Supplementary Table 1). Across plant parts, the total nymphal period of *L. erysimi* significantly varied on test nuclear backgrounds (*F* = 61.93; *df* = 10,90; *P* < 0.001), across cytoplasms (*F* = 12.55; *df* = 3,90; *P* < 0.001), and for the nuclear background × cytoplasm interactions (*F* = 12.28; *df* = 30,270; *P* < 0.001). Across cytoplasms, the total nymphal period was significantly longer on SEJ 8, NPJ 161, LES 39, and NPJ 93 as compared to other nuclear backgrounds (Table 2). Across nuclear backgrounds, the total nymphal period was significantly longer on *ber* as compared to other cytoplasms (Figure 2). Across genotypes and cytoplasms, the total nymphal period was significantly longer on SEJ 8 in *ber* and *juncea*, and on LES 39 in *eru* and *mori* cytoplasms (Table 2).

**TABLE 2:** Effects of cytoplasms and nuclear backgrounds on developmental and reproductive periods of *Lipaphis erysimi* in auto- and alloplasmic lines of *Brassica juncea.*

Nuclear backgrounds (*B. juncea*)	Total nymphal period (h)					Reproductive period (h)					Total developmental period (h)				
	*ber*	*eru*	*mori*	*juncea*	Mean	*ber*	*eru*	*mori*	*juncea*	Mean	*ber*	*eru*	*mori*	*juncea*	Mean
Laxmi	95.1	96.2	88.6	95.0	93.7c	133.1	135.6	137.5	127.0	133.3a	286.0	282.8	286.4	279.1	283.6b
LES 39	101.3	104.4	101.3	93.2	100.1e	149.9	136.0	134.1	161.8	145.5d	318.3	300.2	296.7	320.9	309.0e
NPJ 112	95.8	104.1	95.1	82.1	94.3d	138.0	131.1	132.5	143.7	136.3b	291.5	299.4	287.6	288.9	291.9c
NPJ 139	85.1	83.9	84.1	86.1	84.8a	130.9	143.0	130.0	128.7	133.2a	263.1	280.0	285.0	271.2	274.8a
NPJ 161	100.5	100.7	97.0	100.8	99.8e	137.7	138.6	150.1	131.5	139.5c	302.6	302.8	316.4	296.7	304.6d
NPJ 93	102.2	89.3	98.2	94.8	96.1d	145.5	137.1	133.8	141.8	139.6c	307.3	284.3	288.9	295.1	293.9c
PM 30	89.9	88.7	97.4	91.8	92.0c	133.1	143.4	135.9	133.6	136.5b	273.0	284.7	294.6	281.5	283.5b
Pusa Agrani	94.9	93.5	96.1	92.2	94.2c	144.9	139.2	168.1	132.5	146.2d	300.5	305.6	339.2	278.6	306.0d
Pusa Kisan	97.5	82.5	89.3	84.2	88.4b	138.6	127.0	123.5	139.4	132.1a	289.1	263.0	270.0	281.6	275.9a
Pusa Tarak	82.1	83.6	85.7	91.1	85.6a	135.4	141.4	139.2	128.4	136.1b	276.5	280.0	287.6	286.6	282.7b
SEJ 8	108.3	94.2	94.2	103.6	100.1e	158.3	138.7	132.6	175.3	151.2e	333.6	281.9	284.4	346.0	311.5e
Mean	95.7b	92.8a	93.4a	92.3a		140.5b	137.4a	137.9a	140.3b		294.7b	287.7a	294.3b	293.3b	
For comparing	F-probability		LSD (*P* = 0.05)			F-probability		LSD (*P* = 0.05)			F-probability		LSD (*P* = 0.05)		
Genotypes (G)	<0.001		2.22			<0.001		2.37			<0.001		3.65		
Cytoplasm (C)	<0.001		1.34			<0.001		1.44			<0.001		2.20		
G × C	<0.001		4.45			<0.001		4.77			<0.001		7.29		

The mean values in a row for a parameter following different letters are significant at P = 0.05. The mean values in a column for a parameter following different letters are significant at P = 0.05.

#### Reproductive period

The reproductive period of *L. erysimi* varied from 117.6 to 170.5 h on the leaves, 120.6 to174.5 h on buds and 131.4 to 180.9 h on the siliquae, and varied significantly on the test *B. juncea* nuclear backgrounds, across cytoplasms, and the nuclear backgrounds × cytoplasm interactions (Supplementary Table 2). Across plant parts, the reproductive period of *L. erysimi* significantly varied on the test genotypes (*F* = 60.04; *df* = 10,90; *P* < 0.001), across cytoplasms (*F* = 16.33; *df* = 3,90; *P* < 0.001), and for the nuclear background × cytoplasm interactions (*F* = 41.92; *df* = 30,270; *P* < 0.001). Across cytoplasms, the reproductive period was significantly longer on SEJ 8, NPJ 161, LES 39, NPJ 93, and Pusa Agrani as compared to other nuclear backgrounds (Table 2). Across nuclear backgrounds, the reproductive period was significantly longer on *ber* and *juncea* as compared to other cytoplasms (Figure 2). Across nuclear backgrounds and cytoplasms, the reproductive period was significantly longer on SEJ 8 in *ber* and *juncea*, on NPJ 139, PM 30 and Pusa Tarak in *eru*, and on Pusa Agrani in *mori* cytoplasmic backgrounds (Table 2).

#### Total developmental period

The total developmental period of *L. erysimi* varied from 244.2 to 333.1 h on the leaves, 263.7 to 347.4 h on buds and 280.6 to 363.4 h on the siliquae, and there were significant differences for the *B. juncea* nuclear backgrounds, across cytoplasms, and the nuclear background × cytoplasm interactions (Supplementary Table 3). Across plant parts, there were significant differences in the total developmental period of *L. erysimi* on nuclear backgrounds (*F* = 128.65; *df* = 10,90; *P* < 0.001), across cytoplasms (*F* = 26.54; *df* = 3,90; *P* < 0.001), and for the nuclear background × cytoplasm interactions (*F* = 40.44; *df* = 30,270; *P* < 0.001). Across cytoplasms, the total developmental period was significantly longer on SEJ 8, NPJ 161, LES 39, NPJ 93, and Pusa Agrani as compared to other nuclear backgrounds (Table 2). Across nuclear backgrounds, the total developmental period was significantly lower on *eru* as compared to other cytoplasms (Figure 2). Across nuclear backgrounds and cytoplasms, the total developmental period was significantly greater on SEJ 8 and LES 39 in *ber* and *juncea*, and on Pusa Agrani and NPJ 161 in *eru* and *mori* cytoplasmic backgrounds (Table 2).

#### Reproductive potential

**FIGURE 3:**
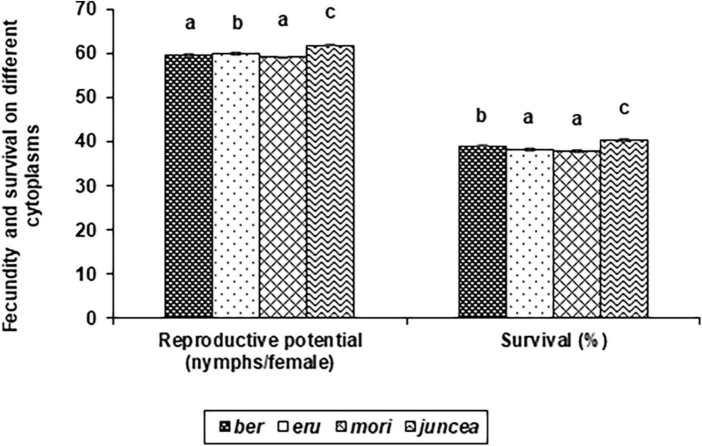
Effects of cytoplasms on reproductive potential and survival *Lipaphis erysimi* in *Brassica juncea.*

The reproductive potential of *L. erysimi* varied from 43.9 to 74.0 nymphs/female on the leaves, 44.6 to 76.6 nymphs/female on buds and 37.2 to 66.1 nymphs/female on the siliquae, and varied significantly on the test *B. juncea* nuclear backgrounds, across cytoplasms, and the nuclear background × cytoplasm interactions (Supplementary Table 4). Across plant parts, there was significant variation in the reproductive potential of *L. erysimi* on the nuclear backgrounds (*F* = 59.33; *df* = 10,90; *P* < 0.001), across cytoplasms (*F* = 23.67; *df* = 3,90; *P* < 0.001), and for the nuclear background × cytoplasm interactions (*F* = 24.07; *df* = 30,270; *P* < 0.001). Across cytoplasms, the reproductive potential was significantly lower on PM 30, Pusa Tarak, SEJ 8, Pusa Kisan, NPJ 93 and Laxmi as compared to other genotypes (Table 3). Across genotypes, the reproductive potential was significantly lower on *ber* and *mori* as compared to other cytoplasms (Figure 3). Across nuclear backgrounds and cytoplasms, the reproductive potential was significantly lower on Pusa Agrani, Pusa Tarak and NPJ 93 in *ber*, PM 30 in *eru* and *mori*, and Pusa Tarak in *juncea* cytoplasmic backgrounds (Table 3).

**TABLE 3:** Effects of cytoplasms and nuclear backgrounds on reproductive potential and survival of *Lipaphis erysimi* in auto- and alloplasmic lines of *Brassica juncea.*

Nuclear backgrounds (*B. juncea*)	Reproductive potential (nymphs/female)					Nymphal survival (%) till 48 h after birth				
	*ber*	*eru*	*mori*	*juncea*	Mean	*ber*	*eru*	*mori*	*juncea*	Mean
Laxmi	59.0	61.8	58.4	58.1	59.3c	35.7	38.4	37.4	35.0	36.6b
LES 39	59.6	62.2	60.7	63.8	61.6d	36.4	38.4	36.2	41.2	38.1c
NPJ 112	59.6	65.3	67.1	59.3	62.8d	42.6	46.3	45.1	39.8	43.5f
NPJ 139	69.3	66.7	60.5	62.4	64.7e	51.0	49.0	43.6	42.8	46.6h
NPJ 161	57.8	63.7	63.3	63.7	62.1d	33.9	39.7	40.2	38.7	38.1c
NPJ 93	56.8	57.0	56.7	62.0	58.1c	38.0	38.8	40.4	41.0	39.6d
PM 30	65.2	45.8	41.9	60.4	53.3a	47.4	17.7	15.1	41.4	30.4a
Pusa Agrani	55.7	66.1	71.5	71.3	66.2f	34.3	46.2	51.2	48.6	45.1g
Pusa Kisan	58.0	56.6	57.2	62.2	58.5c	41.6	40.2	42.3	42.6	41.7e
Pusa Tarak	56.1	56.7	57.0	53.7	55.9b	31.6	30.2	32.1	29.0	30.7a
SEJ 8	57.2	58.6	55.7	64.1	58.9c	37.6	35.9	33.7	44.8	38.0c
Mean	59.5a	60.0b	59.1a	61.9c		39.1b	38.3a	37.9a	40.4b	
For comparing	F-probability		LSD (*P* = 0.05)			F-probability		LSD (*P* = 0.05)		
Genotypes (G)	<0.001		1.47			<0.001		1.32		
Cytoplasm (C)	<0.001		0.88			<0.001		0.79		
G × C	<0.001		2.94			<0.001		2.62		

The mean values in a row for a parameter following different letters are significant at P = 0.05. The mean values in a column for a parameter following different letters are significant at P = 0.05.

#### Survival

The survival of *L. erysimi* varied from 17.9 to 60.0% on the leaves, 17.5 to 60.0% on buds and 9.9 to 41.0% on the siliquae, and there were significant differences for the test *B. juncea* nuclear backgrounds, across cytoplasms, and the nuclear background × cytoplasm interactions (Supplementary Table 5). Across plant parts, there were significant differences in the survival of L. erysimi on test nuclear backgrounds (*F* = 135.66; *df* = 10,90; *P* < 0.001), across cytoplasms (*F* = 30.47; *df* = 3,90; *P* < 0.001), and for the nuclear background × cytoplasm interactions (*F* = 46.14; *df* = 30,270; *P* < 0.001). Across cytoplasms, the *L. erysimi* survival was significantly lower on PM 30, Pusa Tarak and SEJ 8 as compared to other nuclear backgrounds (Table 3). Across nuclear backgrounds, the *L. erysimi* survival was significantly lower on *ber* and *mori* as compared to other cytoplasms (Figure 3). Across nuclear backgrounds and cytoplasms, the *L. erysimi* survival was significantly lower on Pusa Tarak in *ber* and *juncea*, and PM 30 in *eru* and *mori* cytoplasmic backgrounds (Table 3).

### Glucosinolates and myrosinase content in different plant parts of A and B lines of *Brassica juncea*

#### Glucosinolates

**FIGURE 4:**
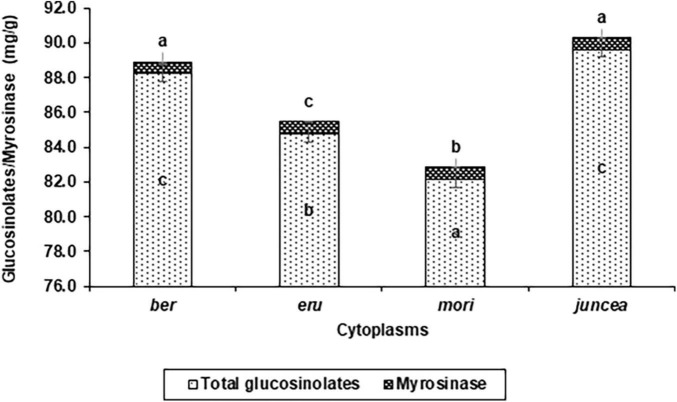
Effects of cytoplasms on total glucosinolate and myrosinase contents in *Brassica juncea.*

The total glucosinolates varied from 29.5 to 77.5 mg/g in the leaves, 66.8 to 109.6 mg/g in buds and 84.6 to 127.4 mg/g in the siliquae, and significantly varied for the test *B. juncea* nuclear backgrounds, across cytoplasms, and the nuclear background × cytoplasm interactions (Supplementary Table 6). Across plant parts, the total glucosinolates significantly varied in the test nuclear backgrounds (*F* = 90.41; *df* = 10,90; *P* < 0.001), across cytoplasms (*F* = 69.59; *df* = 3,90; *P* < 0.001), and for the nuclear background × cytoplasm interactions (*F* = 35.83; *df* = 30,270; *P* < 0.001). Across cytoplasms, the total glucosinolates were significantly higher in Pusa Agrani, SEJ 8, LES 39, PM 30, and NPJ 112 as compared to other nuclear backgrounds (Table 4). Across nuclear backgrounds, the total glucosinolates were significantly higher in *ber* and *juncea* as compared to other cytoplasms (Figure 4). Across genotypes and cytoplasms, the total glucosinolates were significantly higher in SEJ 8, PM 30 and NPJ 112 in *ber*, LES 39, Pusa Agrani and NPJ 112 in *eru* and *mori*, and NPJ 161 and Pusa Agrani in *juncea* cytoplasmic backgrounds (Table 4).

**TABLE 4:** Effects of cytoplasms and nuclear backgrounds on total glucosinolate and myrosinase contents in auto- and alloplasmic lines of *Brassica juncea.*

Nuclear backgrounds (*B. juncea*)	Total glucosinolates (mg/g)					Myrosinase content (mg/g)				
	*ber*	*eru*	*mori*	*juncea*	Mean	*ber*	*eru*	*mori*	*juncea*	Mean
Laxmi	90.3	86.1	61.2	92.5	82.5c	0.58	0.71	0.96	0.65	0.73b
LES 39	88.4	98.7	89.3	84.1	90.1f	0.43	0.59	0.76	0.50	0.57a
NPJ 112	93.4	90.3	89.2	90.0	90.7f	0.60	0.57	0.67	0.55	0.60a
NPJ 139	84.6	76.4	88.7	69.7	79.9b	0.58	0.90	0.63	0.86	0.74b
NPJ 161	88.7	84.3	71.0	103.6	86.9e	0.77	0.97	0.79	0.56	0.77c
NPJ 93	83.7	85.0	78.4	91.7	84.7d	0.87	0.75	0.66	0.66	0.74b
PM 30	94.9	87.8	88.1	86.7	89.4f	0.66	0.62	0.60	0.93	0.70b
Pusa Agrani	87.1	92.8	94.2	101.9	94.0g	0.62	0.47	0.47	0.63	0.55a
Pusa Kisan	73.5	62.4	70.1	77.1	70.8a	0.91	1.04	0.92	0.84	0.93d
Pusa Tarak	90.0	82.6	87.1	94.6	88.6e	0.67	0.66	0.39	0.72	0.61a
SEJ 8	96.1	85.7	86.2	94.0	90.5f	0.45	0.66	0.75	0.45	0.58a
Mean	88.2c	84.7b	82.1a	89.6d		0.65a	0.72b	0.69a	0.67a	
For comparing	F-probability		LSD (*P* = 0.05)			F-probability		LSD (*P* = 0.05)		
Genotypes (G)	<0.001		2.10			<0.001		0.06		
Cytoplasm (C)	<0.001		1.27			<0.001		0.04		
G × C	<0.001		4.20			<0.001		0.13		

The mean values in a row for a parameter following different letters are significant at P = 0.05. The mean values in a column for a parameter following different letters are significant at P = 0.05.

#### Myrosinase

The myrosinase content varied from 0.31 to 0.92 mg/g in the leaves, 0.44 to 1.45 mg/g in buds and 0.38 to 0.99 mg/g in the siliquae, and there were significant differences for the *B. juncea* nuclear backgrounds, across cytoplasms, and the nuclear backgrounds × cytoplasm interactions (Supplementary Table 7). Across plant parts, the myrosinase content significantly varied in the test *B. juncea* nuclear backgrounds (*F* = 39.07; *df* = 10,90; *P* < 0.001), across cytoplasms (*F* = 7.36; *df* = 3,90; *P* < 0.001), and for the nuclear backgrounds × cytoplasm interactions (*F* = 10.39; *df* = 30,270; *P* < 0.001). Across cytoplasms, the myrosinase content was significantly lower in Pusa Agrani, LES 39, SEJ 8, NPJ 112, and Pusa Tarak as compared to other nuclear backgrounds (Table 4). Across genotypes, the myrosinase content was significantly lower in *ber* and *juncea* as compared to other cytoplasms (Figure 4). Across genotypes and cytoplasms, the myrosinase content was significantly lower in LES 39 and SEJ 8 in *ber* and *juncea*, Pusa Agrani, LES 39 and NPJ 112 in *eru*, and Pusa Agrani and Pusa Tarak in *mori* cytoplasmic backgrounds (Table 4).

## Discussion

The parthenogenetic viviparity, high fecundity, and host specificity support *L. erysimi* to multiply faster on *Brassica* plants. The interaction between olfactory cues like plant volatiles and visual stimuli including photosynthetic pigments are crucial for host plant finding by the aphids (Hardie, 1980; Dilawari and Atwal, 1989; Samal et al., 2022). Due to non-availability of aphid resistance sources in the primary gene pool of brassicas (Rana, 2005), the only effective and easily available strategy to control this pest is through systemic insecticides (El-Wakeil et al., 2013). The efforts are going on to increase the yield potential and productivity using hybrid technology in *B. juncea* through different male sterility-inducing cytoplasmic sources derived from the wild brassicas (Chamola et al., 2013). Earlier studies have found that the genetic background of CMS, cytoplasmic factors, and their interaction with nuclear genes influence the morphological, agronomic and physiological traits, and expression of resistance to insect pests and diseases (Dhillon et al., 2008). Present findings revealed that the numbers of aphids/plant, aphid multiplication rate and aphid resistance index were significantly lower on the native cytoplasm of *B. juncea* as compared to male sterile cytoplasms both under natural and artificial infestation conditions. Earlier studies also reported lower oviposition and damage on the maintainer than the CMS lines of sorghum by *Atherigona soccata* (Rondani), *Stenodiplosis sorghicola* (Coquillett), *Peregrinus maidis* Ashmead and *Melanaphis sacchari* Zehnter (Sharma et al., 2004; Dhillon et al., 2006b). Among the male sterile cytoplasms, numbers of aphids/plant, aphid multiplication rate and aphid resistance index were lower for *ber* under natural and for *mori* under artificial infestation conditions as compared to other cytoplasms, indicating that the *ber* and *mori* male sterile cytoplasms have a similar reaction against *L. erysimi*. Varying levels of damage and antixenosis by *A. soccata* in sorghum genotypes under different CMS backgrounds have also been reported earlier, being lower in A4G1 and A4M as compared to other male sterile cytoplasms (Dhillon et al., 2005, 2006a). Furthermore, the numbers of aphids/plant, aphid multiplication rate and aphid resistance index were significantly lower on NPJ 93 in *ber*, on LES 39 and Pusa Agrani in *eru*, and on NPJ 139, PM 30 and NPJ 93 in *mori* cytoplasmic backgrounds under different testing conditions. These findings suggest that not only the CMS source but the cytoplasmic and nuclear genome interactions also play an important role in expressing reaction against *L. erysimi*.

Prolonged developmental periods, and reduced reproduction and survival are important components of the antibiosis mechanism of resistance against *L. erysimi* in *B. juncea* (Samal et al., 2021). Earlier studies reported greater deleterious effects of maintainer cytoplasm on the developmental biology of several insects such as *A. soccata*, *S. sorghicola*, *P. maidis*, and *M. sacchari* (Sharma et al., 1994; Sharma, 2001; Dhillon et al., 2005, 2006a,^2008^) and diseases (Xu and Song, 1997; Xu et al., 1998) as compared to the male sterile cytoplasms, which could be because of interaction between cytoplasmic and nuclear genes of particular CMS line or incomplete genome recovery of the maintainer into CMS lines. Moreover, as a consequence of natural selection pressure, plant species evolved for resisting insect pest’s establishment and multiplication to improve their fitness and adaptability. It was observed in the present study that across cytoplasms, total nymphal, reproductive and total developmental periods were significantly longer on SEJ 8, NPJ 161, LES 39, and NPJ 93, while reproductive potential and survival were lower on PM 30, Pusa Tarak and SEJ 8 as compared to other nuclear backgrounds. Whereas, total nymphal, reproductive and total developmental periods across nuclear backgrounds were significantly longer on *ber*, while reproductive potential and survival were lower on *ber* and *mori* as compared to other cytoplasms, suggesting that the use of these male-sterile cytoplasms in the development of *B. juncea* hybrids could be useful for reducing losses caused by *L. erysimi* in addition to genetic superiority for productivity traits. Earlier studies have also found significant detrimental effects of male sterile cytoplasms like A4M and A4VzM on the development and survival of *A. soccata* in sorghum (Dhillon et al., 2005, 2006a). Further, the total nymphal, reproductive and total developmental periods were significantly longer on SEJ 8 and reproductive potential and survival lower on Pusa Tarak in *ber* and *juncea* cytoplasmic backgrounds as compared to other cytoplasms. The total nymphal period, on the other hand, was significantly higher on LES 39 and total developmental period on Pusa Agrani and NPJ 161 in *eru* and *mori*, while the reproductive period was higher on NPJ 139, PM 30 and Pusa Tarak in *eru*, and Pusa Agrani in *mori* cytoplasmic backgrounds. The reproductive potential and survival were significantly lower on PM 30 in *eru* and *mori* cytoplasmic backgrounds. These findings further reiterate that the CMS as well as cytoplasmic and nuclear gene interactions determine reaction against *L. erysimi*. Earlier studies also found the influence of cytoplasmic factors on the expression of midge and shoot fly resistance in sorghum because of interaction of cytoplasmic and nuclear genes (Sharma et al., 1996; Dhillon et al., 2006c).

Similar to insect reactions, CMS has also been reported to influence the synthesis and metabolism of certain proteins, amino acids, nucleic acid and carbohydrates resulting in altered pollen development and physiology in several crops (Fukasawa, 1957; Erickson, 1967; Savchenko et al., 1968; Alam and Sandal, 1969; Mian et al., 1974; Chen and Qin, 1989; Shi et al., 1996; Murty et al., 1997). Present studies found that across cytoplasms, the total glucosinolates were significantly greater and myrosinase content lower in Pusa Agrani, SEJ 8, LES 39, PM 30, NPJ 112, and Pusa Tarak as compared to other genotypes. Across nuclear backgrounds, the total glucosinolates were significantly higher and myrosinase content lower in *ber* and *juncea* as compared to other cytoplasms. It is already established through earlier studies that the activity of several enzymes like cytochrome oxidase, peroxidase, esterase, ribulose-bisphosphate carboxylase and adenosine triphosphatase is being altered in the anthers of CMS as compared to maintainer lines in several crops (Anonymous, 1977; Watson et al., 1977; Senthil and Manickam, 1995; Murty et al., 1997). Furthermore, it has also been reported that the glucosinolates, a group of isothiocyanates present in the *Brassicas* play a key role in regulating the infestation, establishment and reproduction of aphids (Fahey et al., 2001; Brader et al., 2006; Kumar and Sangha, 2017). In response to herbivore attack, the enzyme myrosinase comes in contact with its glucosinolate substrates, resulting in the production of bioactive compounds (Andreasson et al., 2001). In the present studies, the total glucosinolates were significantly higher and myrosinase content lower in SEJ 8 in *ber* and *B. juncea*, and LES 39 and NPJ 112 in *eru* and *mori* cytoplasmic backgrounds; suggesting that not only the cytoplasmic or nuclear genes but their interaction also regulate the expression of glucosinolates and associated enzymes. These findings will be helpful in the identification of suitable *L. erysimi* tolerant nucleo-cytoplasmic combinations for their deployment in *B. juncea* hybrid breeding program.

## Data availability statement

The original contributions presented in the study are included in the article/Supplementary material, further inquiries can be directed to the corresponding author/s.

## Author contributions

NS and MD conceptualized and designed the study. Both authors performed the experimental setup, data collection, analysis, and wrote and approved the final manuscript.
