# Supplementary material for: Nucleo-cytoplasmic interactions affecting biological performance of Lipaphis erysimi in Brassica juncea
Source: Front Plant Sci. 2022 Aug 18;13:971606. doi: 10.3389/fpls.2022.971606 (PMC9433990; doi:10.3389/fpls.2022.971606)
Supplement: Supplementary file 1 [file Data_Sheet_1.docx]

Supplementary Table 1. Effects of cytoplasms and nuclear backgrounds on total nymphal period of *Lipaphis erysimi* in different plant parts of auto- and alloplasmic lines of *Brassica juncea*

| Nuclear backgrounds (*B. juncea*) | Total nymphal period of *Lipaphis erysimi* on different plant parts (h) | | | | | | | | | | | | | | |
| --- | --- | --- | --- | --- | --- | --- | --- | --- | --- | --- | --- | --- | --- | --- | --- |
|  | Leaves | | | | | Buds | | | | | Siliquae | | | | |
|  | *ber* | *eru* | *mori* | *juncea* | Mean | *ber* | *eru* | *mori* | *juncea* | Mean | *ber* | *eru* | *mori* | *juncea* | Mean |
| Laxmi | 88.9 | 90.8 | 83.8 | 90.4 | 88.5c | 98.9 | 98.4 | 91.6 | 97.8 | 96.7c | 97.4 | 99.3 | 90.3 | 96.6 | 95.9c |
| LES 39 | 91.7 | 100.1 | 90.9 | 75.2 | 89.5d | 103.1 | 103.3 | 102.3 | 99.9 | 102.2 | 109.1 | 109.9 | 110.8 | 104.5 | 108.6f |
| NPJ 112 | 90.1 | 99.7 | 90.6 | 77.2 | 89.4c | 99.5 | 107.2 | 98.1 | 83.5 | 97.1c | 97.9 | 105.3 | 96.4 | 85.5 | 96.3c |
| NPJ 139 | 80.4 | 79.5 | 79.8 | 81.5 | 80.3a | 88.1 | 87.1 | 87.1 | 89.1 | 87.9a | 87.0 | 85.2 | 85.5 | 87.8 | 86.4a |
| NPJ 161 | 90.8 | 88.6 | 92.0 | 87.7 | 89.8d | 101.8 | 103.4 | 95.1 | 104.3 | 101.2d | 108.9 | 110.2 | 104.0 | 110.3 | 108.4f |
| NPJ 93 | 84.7 | 79.6 | 93.9 | 90.9 | 87.3c | 107.3 | 89.0 | 97.2 | 88.6 | 95.5c | 114.6 | 99.4 | 103.6 | 104.9 | 105.6e |
| PM 30 | 84.4 | 84.1 | 91.8 | 78.9 | 84.8b | 90.1 | 90.2 | 102.0 | 96.3 | 94.7c | 95.4 | 91.8 | 98.2 | 100.2 | 96.4c |
| Pusa Agrani | 90.8 | 88.5 | 91.9 | 86.9 | 89.5d | 98.3 | 96.1 | 99.5 | 95.4 | 97.3c | 95.5 | 96.0 | 97.0 | 94.2 | 95.7c |
| Pusa Kisan | 93.0 | 78.2 | 84.3 | 80.4 | 84.0b | 100.9 | 86.0 | 91.9 | 87.1 | 91.5b | 98.7 | 83.4 | 91.6 | 85.0 | 89.7b |
| Pusa Tarak | 78.5 | 79.2 | 82.1 | 85.4 | 81.3a | 84.8 | 86.4 | 89.4 | 93.7 | 88.6a | 83.0 | 85.2 | 85.7 | 94.3 | 87.1a |
| SEJ 8 | 104.2 | 90.5 | 89.0 | 99.7 | 95.9e | 112.0 | 96.1 | 98.6 | 107.3 | 103.5d | 108.6 | 95.9 | 95.0 | 103.9 | 100.9d |
| Mean | 88.9c | 87.2b | 88.2bc | 84.9a |  | 98.6b | 94.8a | 95.7a | 94.8a |  | 99.6b | 96.5a | 96.2a | 97.0a |  |
| For comparing | F-probability | | LSD (P = 0.05) | | | F-probability | | LSD (P = 0.05) | | | F-probability | | LSD (P = 0.05) | | |
| Genotypes (G) | <0.001 | | 2.12 | | | <0.001 | | 2.56 | | | <0.001 | | 1.99 | | |
| Cytoplasm (C) | <0.001 | | 1.28 | | | <0.001 | | 1.55 | | | <0.001 | | 1.20 | | |
| G × C | <0.001 | | 4.24 | | | <0.001 | | 5.12 | | | <0.001 | | 3.99 | | |

The mean values in a row for a parameter following different letters are significant at P = 0.05. The mean values in a column for a parameter following different letters are significant at P = 0.05.

Supplementary Table 2. Effects of cytoplasms and nuclear backgrounds on reproductive period of *Lipaphis erysimi* in different plant parts of auto- and alloplasmic lines of *Brassica juncea*

| Nuclear backgrounds (*B. juncea*) | Reproductive period of *Lipaphis erysimi* on different plant parts (h) | | | | | | | | | | | | | | | | | | | |
| --- | --- | --- | --- | --- | --- | --- | --- | --- | --- | --- | --- | --- | --- | --- | --- | --- | --- | --- | --- | --- |
|  | Leaves | | | | | | | Buds | | | | | | | Siliquae | | | | | |
|  | *ber* | *eru* | *mori* | | *juncea* | Mean | *ber* | | *eru* | *mori* | | *juncea* | Mean | *ber* | | *eru* | *mori* | | *juncea* | Mean |
| Laxmi | 127.2 | 133.2 | 131.4 | | 121.2 | 128.3a | 131.2 | | 139.2 | 136.4 | | 125.2 | 133.0a | 140.9 | | 134.3 | 144.6 | | 134.7 | 138.6a |
| LES 39 | 137.8 | 129.2 | 128.4 | | 140.4 | 134.0c | 148.2 | | 133.2 | 130.8 | | 165.5 | 144.4c | 163.6 | | 145.5 | 143.1 | | 179.4 | 157.9g |
| NPJ 112 | 134.8 | 126.2 | 125.4 | | 137.4 | 131.0b | 135.8 | | 127.2 | 131.4 | | 140.4 | 133.7b | 143.3 | | 139.8 | 140.8 | | 153.4 | 144.3c |
| NPJ 139 | 126.0 | 138.0 | 123.6 | | 122.4 | 127.5a | 131.0 | | 142.0 | 127.6 | | 126.4 | 131.8a | 135.8 | | 148.9 | 138.8 | | 137.2 | 140.2b |
| NPJ 161 | 144.4 | 132.1 | 165.8 | | 135.0 | 144.3e | 127.2 | | 136.2 | 135.4 | | 124.2 | 130.8a | 141.5 | | 147.5 | 149.2 | | 135.4 | 143.4c |
| NPJ 93 | 135.4 | 137.4 | 137.8 | | 136.2 | 136.7c | 145.8 | | 131.2 | 120.6 | | 134.4 | 133.0a | 155.4 | | 142.8 | 143.1 | | 154.8 | 149.0e |
| PM 30 | 121.6 | 137.2 | 131.7 | | 124.2 | 128.7a | 132.0 | | 142.2 | 132.7 | | 131.6 | 134.6b | 145.6 | | 150.7 | 143.2 | | 145.1 | 146.2d |
| Pusa Agrani | 141.4 | 129.1 | 162.8 | | 129.0 | 140.6d | 143.4 | | 134.1 | 166.1 | | 133.0 | 144.2c | 149.8 | | 154.5 | 175.5 | | 135.5 | 153.8f |
| Pusa Kisan | 135.8 | 120.2 | 117.6 | | 134.4 | 127.0a | 140.8 | | 126.2 | 121.6 | | 138.4 | 131.8a | 139.1 | | 134.5 | 131.4 | | 145.4 | 137.6a |
| Pusa Tarak | 130.2 | 136.2 | 134.4 | | 124.2 | 131.3b | 133.2 | | 143.2 | 138.4 | | 124.2 | 134.8b | 142.7 | | 144.7 | 144.9 | | 136.7 | 142.3b |
| SEJ 8 | 152.2 | 134.2 | 127.8 | | 170.5 | 146.2e | 156.2 | | 137.2 | 131.8 | | 174.5 | 149.9d | 166.6 | | 144.6 | 138.2 | | 180.9 | 157.6g |
| Mean | 135.2b | 132.1a | 135.2b | | 134.1b |  | 138.6c | | 135.6b | 133.9a | | 138.0c |  | 147.7b | | 144.3a | 144.8a | | 149.0b |  |
| For comparing | F-probability | | | LSD (P = 0.05) | | | | F-probability | | | LSD (P = 0.05) | | | | F-probability | | | LSD (P = 0.05) | | |
| Genotypes (G) | <0.001 | | | 2.71 | | | | <0.001 | | | 2.40 | | | | <0.001 | | | 2.00 | | |
| Cytoplasm (C) | <0.001 | | | 1.63 | | | | <0.001 | | | 1.50 | | | | <0.001 | | | 1.20 | | |
| G × C | <0.001 | | | 5.41 | | | | <0.001 | | | 4.90 | | | | <0.001 | | | 4.00 | | |

The mean values in a row for a parameter following different letters are significant at P = 0.05. The mean values in a column for a parameter following different letters are significant at P = 0.05.

Supplementary Table 3. Effects of cytoplasms and nuclear backgrounds on total developmental period of *Lipaphis erysimi* in different plant parts of auto- and alloplasmic lines of *Brassica juncea*

| Nuclear backgrounds (*B. juncea*) | Total developmental period of *Lipaphis erysimi* on different plant parts (h) | | | | | | | | | | | | | | |
| --- | --- | --- | --- | --- | --- | --- | --- | --- | --- | --- | --- | --- | --- | --- | --- |
|  | Leaves | | | | | Buds | | | | | Siliquae | | | | |
|  | *ber* | *eru* | *mori* | *juncea* | Mean | *ber* | *eru* | *mori* | *juncea* | Mean | *ber* | *eru* | *mori* | *juncea* | Mean |
| Laxmi | 266.1 | 270.4 | 264.7 | 266.1 | 266.8d | 287.8 | 288.8 | 285.8 | 281.2 | 285.9b | 304.2 | 289.2 | 308.8 | 289.9 | 298.0a |
| LES 39 | 285.7 | 286.7 | 274.3 | 271.8 | 279.6g | 313.4 | 294.4 | 293.8 | 327.5 | 307.3d | 355.8 | 319.5 | 322.1 | 363.4 | 340.2e |
| NPJ 112 | 278.8 | 281.5 | 269.4 | 271.6 | 275.3f | 291.1 | 300.0 | 295.0 | 287.4 | 293.4c | 304.6 | 316.7 | 298.3 | 307.6 | 306.8b |
| NPJ 139 | 244.2 | 258.4 | 264.9 | 248.2 | 253.9a | 263.6 | 277.6 | 282.9 | 268.3 | 273.1a | 281.4 | 303.9 | 307.3 | 297.2 | 297.5a |
| NPJ 161 | 287.6 | 289.9 | 327.2 | 271.9 | 294.2i | 292.7 | 292.8 | 292.6 | 293.0 | 292.8c | 327.5 | 325.7 | 329.4 | 325.1 | 326.9d |
| NPJ 93 | 269.3 | 265.9 | 276.1 | 275.3 | 271.7e | 312.1 | 270.0 | 278.3 | 283.5 | 286.0b | 340.6 | 316.9 | 312.4 | 326.5 | 324.1c |
| PM 30 | 252.7 | 268.9 | 276.8 | 250.5 | 262.2c | 269.6 | 286.3 | 296.8 | 282.9 | 283.9b | 296.7 | 299.0 | 310.2 | 311.2 | 304.3b |
| Pusa Agrani | 284.8 | 285.7 | 324.1 | 265.6 | 290.1h | 300.9 | 302.0 | 338.7 | 280.6 | 305.6d | 315.7 | 329.1 | 354.8 | 289.6 | 322.3c |
| Pusa Kisan | 272.9 | 244.7 | 248.1 | 262.9 | 257.2b | 291.7 | 263.7 | 265.7 | 278.0 | 274.8a | 302.7 | 280.6 | 296.1 | 303.8 | 295.8a |
| Pusa Tarak | 262.6 | 268.6 | 272.6 | 270.8 | 268.7d | 281.7 | 282.5 | 287.7 | 284.0 | 284.0b | 285.1 | 288.8 | 302.4 | 305.0 | 295.3a |
| SEJ 8 | 318.5 | 269.1 | 267.7 | 333.1 | 297.1j | 335.9 | 283.4 | 288.1 | 347.4 | 313.7e | 346.4 | 293.3 | 297.3 | 357.6 | 323.7c |
| Mean | 274.8b | 271.8a | 278.7b | 271.6a |  | 294.6c | 285.6a | 291.4b | 292.2b |  | 314.6c | 305.7a | 312.6b | 316.1c |  |
| For comparing | F-probability | | LSD (P = 0.05) | | | F-probability | | LSD (P = 0.05) | | | F-probability | | LSD (P = 0.05) | | |
| Genotypes (G) | <0.001 | | 3.70 | | | <0.001 | | 4.02 | | | <0.001 | | 3.22 | | |
| Cytoplasm (C) | <0.001 | | 2.23 | | | <0.001 | | 2.42 | | | <0.001 | | 1.94 | | |
| G × C | <0.001 | | 7.40 | | | <0.001 | | 8.04 | | | <0.001 | | 6.44 | | |

The mean values in a row for a parameter following different letters are significant at P = 0.05. The mean values in a column for a parameter following different letters are significant at P = 0.05.

Supplementary Table 4. Effects of cytoplasms and nuclear backgrounds on reproductive potential of *Lipaphis erysimi* in different plant parts of auto- and alloplasmic lines of *Brassica juncea*

| Nuclear backgrounds (*B. juncea*) | Reproductive potential of *Lipaphis erysimi* on different plant parts (nymphs/female) | | | | | | | | | | | | | | |
| --- | --- | --- | --- | --- | --- | --- | --- | --- | --- | --- | --- | --- | --- | --- | --- |
|  | Leaves | | | | | Buds | | | | | Siliquae | | | | |
|  | *ber* | *eru* | *mori* | *juncea* | Mean | *ber* | *eru* | *mori* | *juncea* | Mean | *ber* | *eru* | *mori* | *juncea* | Mean |
| Laxmi | 59.6 | 62.4 | 59.2 | 58.8 | 60.0c | 63.9 | 66.8 | 63.4 | 63.0 | 64.3d | 53.4 | 56.1 | 52.8 | 52.5 | 53.7c |
| LES 39 | 61.4 | 66.7 | 67.6 | 61.0 | 64.2e | 63.9 | 65.3 | 62.5 | 70.7 | 65.6e | 53.5 | 54.7 | 51.9 | 59.7 | 55.0d |
| NPJ 112 | 61.3 | 66.8 | 70.6 | 60.9 | 64.9e | 64.0 | 69.3 | 70.3 | 63.7 | 66.8f | 53.6 | 59.8 | 60.5 | 53.2 | 56.8e |
| NPJ 139 | 74.0 | 71.2 | 66.3 | 67.1 | 69.7g | 72.3 | 69.5 | 64.4 | 65.4 | 67.9g | 61.5 | 59.5 | 50.8 | 54.6 | 56.6e |
| NPJ 161 | 55.6 | 67.9 | 73.3 | 73.9 | 67.7f | 65.6 | 68.5 | 65.2 | 64.9 | 66.1e | 52.1 | 54.8 | 51.4 | 52.3 | 52.7c |
| NPJ 93 | 59.1 | 63.4 | 62.4 | 62.4 | 61.8d | 62.9 | 61.2 | 61.9 | 68.6 | 63.7d | 48.3 | 46.5 | 45.9 | 55.0 | 48.9a |
| PM 30 | 61.0 | 46.9 | 43.9 | 61.3 | 53.3a | 74.1 | 48.6 | 44.6 | 67.3 | 58.7a | 60.4 | 41.8 | 37.2 | 52.7 | 48.0a |
| Pusa Agrani | 55.5 | 67.9 | 73.2 | 71.1 | 66.9f | 60.2 | 70.6 | 76.0 | 76.6 | 70.9h | 51.5 | 59.9 | 65.3 | 66.1 | 60.7f |
| Pusa Kisan | 62.4 | 61.3 | 61.9 | 63.3 | 62.2d | 61.2 | 59.7 | 60.2 | 67.0 | 62.0c | 50.5 | 48.8 | 49.4 | 56.2 | 51.2b |
| Pusa Tarak | 57.8 | 58.5 | 58.7 | 54.0 | 57.3b | 60.5 | 61.0 | 61.4 | 58.6 | 60.4b | 50.1 | 50.7 | 51.0 | 48.3 | 50.0b |
| SEJ 8 | 57.8 | 59.2 | 56.3 | 65.0 | 59.6c | 62.4 | 63.6 | 60.9 | 69.0 | 64.0d | 51.6 | 52.9 | 50.0 | 58.3 | 53.2c |
| Mean | 60.5a | 62.9b | 63.0b | 63.5b |  | 64.6b | 64.0b | 62.8a | 66.8c |  | 53.3b | 53.2b | 51.5a | 55.4c |  |
| For comparing | F-probability | | LSD (P = 0.05) | | | F-probability | | LSD (P = 0.05) | | | F-probability | | LSD (P = 0.05) | | |
| Genotypes (G) | <0.001 | | 1.48 | | | <0.001 | | 1.50 | | | <0.001 | | 1.43 | | |
| Cytoplasm (C) | <0.001 | | 0.89 | | | <0.001 | | 0.90 | | | <0.001 | | 0.86 | | |
| G × C | <0.001 | | 2.96 | | | <0.001 | | 3.00 | | | <0.001 | | 2.85 | | |

The mean values in a row for a parameter following different letters are significant at P = 0.05. The mean values in a column for a parameter following different letters are significant at P = 0.05.

Supplementary Table 5. Effects of cytoplasms and nuclear backgrounds on survival of *Lipaphis erysimi* in different plant parts of auto- and alloplasmic lines of *Brassica juncea*

| Nuclear backgrounds (*B. juncea*) | Survival of *Lipaphis erysimi* on different plant parts (%) | | | | | | | | | | | | | | |
| --- | --- | --- | --- | --- | --- | --- | --- | --- | --- | --- | --- | --- | --- | --- | --- |
|  | Leaves | | | | | Buds | | | | | Siliquae | | | | |
|  | *ber* | *eru* | *mori* | *juncea* | Mean | *ber* | *eru* | *mori* | *juncea* | Mean | *ber* | *eru* | *mori* | *juncea* | Mean |
| Laxmi | 41.4 | 44.1 | 42.4 | 40.6 | 42.1d | 40.8 | 44.4 | 41.7 | 40.0 | 41.7c | 24.9 | 26.8 | 28.0 | 24.3 | 26.0b |
| LES 39 | 37.4 | 41.0 | 39.4 | 40.5 | 39.6c | 42.7 | 44.0 | 41.2 | 49.5 | 44.4d | 29.2 | 30.3 | 28.0 | 33.8 | 30.3d |
| NPJ 112 | 43.2 | 49.2 | 47.8 | 42.3 | 45.6e | 50.1 | 52.3 | 52.4 | 45.2 | 50.0f | 34.4 | 37.4 | 35.2 | 32.0 | 34.8f |
| NPJ 139 | 60.0 | 57.6 | 52.5 | 51.0 | 55.3g | 54.3 | 53.0 | 47.2 | 45.7 | 50.1f | 38.8 | 36.4 | 31.1 | 31.7 | 34.5f |
| NPJ 161 | 29.8 | 42.5 | 47.7 | 44.8 | 41.2d | 43.8 | 46.5 | 44.7 | 43.0 | 44.5d | 28.2 | 30.1 | 28.3 | 28.5 | 28.8c |
| NPJ 93 | 35.7 | 41.1 | 42.2 | 37.3 | 39.1c | 47.7 | 46.0 | 48.3 | 50.1 | 48.0e | 30.5 | 29.4 | 30.9 | 35.5 | 31.6e |
| PM 30 | 41.1 | 20.3 | 17.9 | 40.9 | 30.1a | 60.0 | 20.3 | 17.5 | 50.6 | 37.1b | 41.0 | 12.5 | 9.9 | 32.7 | 24.0a |
| Pusa Agrani | 36.2 | 50.1 | 55.4 | 51.1 | 48.2f | 40.9 | 53.3 | 58.8 | 55.9 | 52.2g | 25.8 | 35.1 | 39.4 | 38.9 | 34.8f |
| Pusa Kisan | 49.4 | 48.0 | 50.4 | 48.2 | 49.0f | 44.6 | 43.0 | 45.2 | 46.9 | 44.9d | 30.8 | 29.5 | 31.4 | 32.6 | 31.1d |
| Pusa Tarak | 33.9 | 32.2 | 34.1 | 28.3 | 32.1b | 36.5 | 34.7 | 36.7 | 35.2 | 35.8a | 24.4 | 23.7 | 25.5 | 23.6 | 24.3a |
| SEJ 8 | 40.3 | 41.6 | 38.8 | 47.4 | 42.0d | 44.4 | 41.0 | 38.3 | 51.4 | 43.8d | 28.0 | 25.1 | 24.1 | 35.5 | 28.2c |
| Mean | 40.8a | 42.5b | 42.6b | 42.9b |  | 46.0b | 43.5a | 42.9a | 46.7b |  | 30.5b | 28.8a | 28.3a | 31.7c |  |
| For comparing | F-probability | | LSD (P = 0.05) | | | F-probability | | LSD (P = 0.05) | | | F-probability | | LSD (P = 0.05) | | |
| Genotypes (G) | <0.001 | | 1.45 | | | <0.001 | | 1.41 | | | <0.001 | | 1.09 | | |
| Cytoplasm (C) | <0.001 | | 0.87 | | | <0.001 | | 0.85 | | | <0.001 | | 0.65 | | |
| G × C | <0.001 | | 2.89 | | | <0.001 | | 2.81 | | | <0.001 | | 2.17 | | |

The mean values in a row for a parameter following different letters are significant at P = 0.05. The mean values in a column for a parameter following different letters are significant at P = 0.05.

Supplementary Table 6. Effects of cytoplasms and nuclear backgrounds on total glucosinolates in different plant parts of auto- and alloplasmic lines of *Brassica juncea*

| Nuclear backgrounds (*B. juncea*) | Total glucosinolates in different plant parts (mg/g) | | | | | | | | | | | | | | |
| --- | --- | --- | --- | --- | --- | --- | --- | --- | --- | --- | --- | --- | --- | --- | --- |
|  | Leaves | | | | | Buds | | | | | Siliquae | | | | |
|  | *ber* | *eru* | *mori* | *juncea* | Mean | *ber* | *eru* | *mori* | *juncea* | Mean | *ber* | *eru* | *mori* | *juncea* | Mean |
| Laxmi | 65.6 | 55.3 | 32.3 | 64.4 | 54.4b | 90.6 | 92.1 | 66.8 | 99.7 | 87.3c | 114.7 | 110.8 | 84.6 | 113.5 | 105.9b |
| LES 39 | 67.2 | 70.2 | 55.5 | 54.9 | 62.0e | 88.2 | 98.5 | 94.9 | 88.6 | 92.6d | 110.0 | 127.4 | 117.5 | 108.8 | 115.9e |
| NPJ 112 | 65.6 | 64.0 | 52.9 | 73.9 | 64.1f | 101.2 | 93.3 | 100.5 | 86.2 | 95.3e | 113.5 | 113.5 | 114.3 | 110.0 | 112.8d |
| NPJ 139 | 64.4 | 45.4 | 68.4 | 41.0 | 54.8b | 88.2 | 79.5 | 88.6 | 67.6 | 81.0b | 101.2 | 104.4 | 109.2 | 100.5 | 103.8b |
| NPJ 161 | 58.1 | 67.2 | 40.2 | 73.9 | 59.9d | 95.3 | 82.2 | 77.5 | 109.6 | 91.2d | 112.7 | 103.6 | 95.3 | 127.4 | 109.8c |
| NPJ 93 | 52.1 | 55.3 | 54.9 | 63.2 | 56.4c | 90.6 | 90.9 | 78.7 | 95.7 | 89.0c | 108.4 | 108.8 | 101.6 | 116.3 | 108.8c |
| PM 30 | 73.9 | 64.4 | 64.0 | 65.2 | 66.9g | 96.9 | 86.2 | 90.2 | 90.6 | 91.0d | 113.9 | 112.7 | 110.0 | 104.4 | 110.3c |
| Pusa Agrani | 56.1 | 77.5 | 64.0 | 70.7 | 67.1g | 92.5 | 90.2 | 102.4 | 108.0 | 98.3f | 112.7 | 110.8 | 116.3 | 127.0 | 116.7e |
| Pusa Kisan | 43.8 | 29.5 | 41.0 | 48.2 | 40.6a | 79.5 | 69.9 | 76.7 | 81.0 | 76.8a | 97.3 | 87.8 | 92.5 | 102.0 | 94.9a |
| Pusa Tarak | 60.8 | 52.9 | 64.4 | 66.0 | 61.0d | 95.7 | 88.6 | 88.2 | 101.2 | 93.4e | 113.5 | 106.4 | 108.8 | 116.7 | 111.4c |
| SEJ 8 | 74.3 | 63.2 | 54.1 | 68.8 | 65.1f | 100.1 | 88.2 | 88.6 | 100.5 | 94.4e | 113.9 | 105.6 | 115.9 | 112.7 | 112.0d |
| Mean | 62.0c | 58.6b | 53.8a | 62.7c |  | 92.6b | 87.2a | 86.6a | 93.5b |  | 110.2c | 108.3b | 106.0a | 112.7d |  |
| For comparing | F-probability | | LSD (P = 0.05) | | | F-probability | | LSD (P = 0.05) | | | F-probability | | LSD (P = 0.05) | | |
| Genotypes (G) | <0.001 | | 1.76 | | | <0.001 | | 2.09 | | | <0.001 | | 2.45 | | |
| Cytoplasm (C) | <0.001 | | 1.06 | | | <0.001 | | 1.26 | | | <0.001 | | 1.48 | | |
| G × C | <0.001 | | 3.51 | | | <0.001 | | 4.19 | | | <0.001 | | 4.91 | | |

The mean values in a row for a parameter following different letters are significant at P = 0.05. The mean values in a column for a parameter following different letters are significant at P = 0.05.

Supplementary Table 7. Effects of cytoplasms and nuclear backgrounds on myrosinase content in different plant parts of auto- and alloplasmic lines of *Brassica juncea*

| Nuclear backgrounds (*B. juncea*) | Myrosinase content in different plant parts (mg/g) | | | | | | | | | | | | | | |
| --- | --- | --- | --- | --- | --- | --- | --- | --- | --- | --- | --- | --- | --- | --- | --- |
|  | Leaves | | | | | Buds | | | | | Siliquae | | | | |
|  | *ber* | *eru* | *mori* | *juncea* | Mean | *ber* | *eru* | *mori* | *juncea* | Mean | *ber* | *eru* | *mori* | *juncea* | Mean |
| Laxmi | 0.54 | 0.64 | 0.84 | 0.52 | 0.64e | 0.65 | 0.84 | 1.16 | 0.80 | 0.86b | 0.55 | 0.64 | 0.89 | 0.63 | 0.68b |
| LES 39 | 0.31 | 0.32 | 0.53 | 0.36 | 0.38a | 0.50 | 0.78 | 1.03 | 0.61 | 0.73a | 0.47 | 0.68 | 0.73 | 0.51 | 0.60a |
| NPJ 112 | 0.39 | 0.47 | 0.55 | 0.45 | 0.47c | 0.76 | 0.67 | 0.80 | 0.65 | 0.72a | 0.66 | 0.57 | 0.67 | 0.55 | 0.61a |
| NPJ 139 | 0.62 | 0.83 | 0.61 | 0.80 | 0.72f | 0.63 | 0.99 | 0.69 | 0.95 | 0.82b | 0.50 | 0.88 | 0.59 | 0.85 | 0.71c |
| NPJ 161 | 0.60 | 0.50 | 0.71 | 0.38 | 0.55d | 0.97 | 1.45 | 0.86 | 0.70 | 1.00d | 0.74 | 0.95 | 0.78 | 0.60 | 0.77c |
| NPJ 93 | 0.64 | 0.59 | 0.46 | 0.47 | 0.54d | 1.10 | 0.88 | 0.84 | 0.80 | 0.91c | 0.86 | 0.79 | 0.68 | 0.70 | 0.76c |
| PM 30 | 0.37 | 0.40 | 0.43 | 0.48 | 0.42b | 0.86 | 0.83 | 0.74 | 1.39 | 0.96c | 0.76 | 0.62 | 0.64 | 0.92 | 0.74c |
| Pusa Agrani | 0.49 | 0.33 | 0.43 | 0.44 | 0.42b | 0.78 | 0.59 | 0.55 | 0.78 | 0.68a | 0.58 | 0.49 | 0.45 | 0.68 | 0.55a |
| Pusa Kisan | 0.78 | 0.92 | 0.77 | 0.71 | 0.80g | 1.03 | 1.22 | 1.07 | 0.96 | 1.07d | 0.93 | 0.99 | 0.92 | 0.84 | 0.92d |
| Pusa Tarak | 0.49 | 0.44 | 0.35 | 0.51 | 0.45b | 0.86 | 0.82 | 0.44 | 0.93 | 0.76a | 0.66 | 0.72 | 0.38 | 0.73 | 0.62b |
| SEJ 8 | 0.40 | 0.57 | 0.57 | 0.43 | 0.49c | 0.53 | 0.78 | 0.93 | 0.53 | 0.69a | 0.43 | 0.65 | 0.76 | 0.39 | 0.56a |
| Mean | 0.51a | 0.55b | 0.57b | 0.50a |  | 0.79a | 0.90b | 0.83a | 0.83a |  | 0.65a | 0.73b | 0.68a | 0.67a |  |
| For comparing | F-probability | | LSD (P = 0.05) | | | F-probability | | LSD (P = 0.05) | | | F-probability | | LSD (P = 0.05) | | |
| Genotypes (G) | <0.001 | | 0.04 | | | <0.001 | | 0.08 | | | <0.001 | | 0.06 | | |
| Cytoplasm (C) | <0.001 | | 0.03 | | | <0.001 | | 0.05 | | | 0.002 | | 0.04 | | |
| G × C | <0.001 | | 0.09 | | | <0.001 | | 0.17 | | | <0.001 | | 0.13 | | |

The mean values in a row for a parameter following different letters are significant at P = 0.05. The mean values in a column for a parameter following different letters are significant at P = 0.05.
